# Short- and long-term outcomes of laparoscopic surgery for colorectal cancer in the elderly aged over 80 years old versus non-elderly: a retrospective cohort study

**DOI:** 10.1186/s12877-020-01779-2

**Published:** 2020-11-04

**Authors:** Yoshitake Ueda, Norio Shiraishi, Takahide Kawasaki, Tomonori Akagi, Shigeo Ninomiya, Hidefumi Shiroshita, Tsuyoshi Etoh, Masafumi Inomata

**Affiliations:** 1 Department of Comprehensive Surgery for Community Medicine, Oita University Faculty of Medicine, Hasama-machi, Oita, 879-5593 Japan; 2 Department of Gastroenterological and Pediatric Surgery, Oita University Faculty of Medicine, Oita, Japan

**Keywords:** Colorectal cancer, Elderly, Laparoscopic surgery, Safety, Curability

## Abstract

**Background:**

Recently, colorectal cancer has increased in elderly patients worldwide, with laparoscopic colorectal surgery increasing in elderly patients with colorectal cancer. However, whether laparoscopic colorectal surgery is an optimal procedure for colorectal cancer in the elderly remains unclear. This study aimed to verify safety and curability of laparoscopic colorectal surgery in elderly patients ≥80 years old.

**Methods:**

Patients undergoing curative colorectal surgery from 2006 to 2014 were enrolled and classified into the laparoscopic surgery in elderly patients aged ≥80 years (LAC-E) group, open surgery in elderly patients (OC-E) group, and laparoscopic surgery in non-elderly patients (LAC-NE) group. Short- and long-term outcomes were compared between these groups.

**Results:**

The LAC-E, OC-E, and LAC-NE groups comprised 85, 25, and 358 patients, respectively. Intraoperative blood loss and incidence of postoperative complications were significantly lower in the LAC-E versus OC-E group (97 vs. 440 mL, *p* < .01 and 14% vs. 32%, *p* < .05, respectively). Long-term outcomes were not different between these two groups. Operation time was significantly shorter in the LAC-E versus LAC-NE group (249 vs. 288 min, *p* < .01). Intraoperative blood loss and postoperative complications were similar between the groups. Although the 5-year overall survival rate in the LAC-E group was lower than that in the LAC-NE group (64% vs. 80%, *p* < .01), there was no difference in 5-year disease-specific survival between the groups.

**Conclusion:**

Laparoscopic colorectal surgery is technically and oncologically safe for colorectal cancer in the elderly as well as the non-elderly and can be an optimal procedure for colorectal cancer in the elderly.

## Background

The population of elderly persons, and especially those in Japan aged over 80 years, have been dramatically increasing worldwide. Colorectal cancer is the third most commonly occurring cancer in men and the second in women worldwide [1]. In Japan, the incidence of colorectal cancer was ranked as the most common cancer type and the second leading cause of cancer-related death in 2019 [2]. Thus, aging could be considered as one of the major risk factors for colorectal carcinogenesis [3]. Consequently, the incidence of colorectal cancer in elderly patients has been increasing year by year in Japan, with more than 25% of colorectal cancer patients now aged 80 years or over [4].

In the treatment of colorectal cancer, laparoscopic surgery has developed rapidly throughout the world due to advantages such as smaller incision, less pain, reduced intraoperative blood loss, faster recovery, and shorter hospitalization compared with open surgery [5–7]. These clinical benefits of laparoscopic surgery for colorectal cancer have been proved by a series of a large sample, multicenter, randomized controlled trials [8–11]. Laparoscopic colorectal surgery has become a standard procedure for treating colorectal cancer worldwide. As a result, the incidence of laparoscopic colorectal surgery in elderly patients has also been increasing in Japan recently [12]. However, the majority of the previous trials of laparoscopic colorectal surgery was conducted on patients younger than 80 years. In addition, most previous studies have reported only the technical safety and short-term outcomes of laparoscopic surgery in comparison with open surgery for elderly patients, but they did not include long-term outcomes. Thus, it is still unclear whether laparoscopic colorectal surgery is an optimal procedure for colorectal cancer in the elderly.

Therefore, we aimed to evaluate the technical and oncological safety of laparoscopic surgery for elderly patients 80 years and older with colorectal cancer by retrospectively comparing their short- and long-term surgical outcomes with those of open surgery for elderly patients and laparoscopic surgery for non-elderly patients aged < 80 years.

## Methods

### Patients

Between April 2006 and June 2014, 468 patients with colorectal cancer who underwent curative surgery in our department were enrolled in this retrospective study. Patients with synchronous metastases and patients who received palliative and emergent operations were excluded from this study. The patients were divided into three groups. The laparoscopic surgery in elderly patients (LAC-E) group included 85 patients aged ≥80 years who underwent laparoscopic surgery. The open surgery in elderly patients (OC-E) group included 25 patients aged ≥80 years who underwent open surgery. The laparoscopic surgery in non-elderly patients (LAC-NE) group included 358 patients aged < 80 years who underwent laparoscopic surgery. Patients’ demographics, preoperative and operative variables, the clinicopathological findings, and postoperative short- and long-term outcomes for all patients were obtained from the patients’ medical records, operation records, and pathology records in our hospital database. The three groups were examined and compared in terms of patient characteristics such as age, sex, American Society of Anesthesiologists physical status (ASA-PS) classification, presence of symptom, previous abdominal surgery, comorbidities, pre- and post-operative chemotherapy, and pathological findings including tumor location, tumor differentiation, tumor size, pTNM stage (Union for International Cancer Control, 7th edition [13]), and short-term outcomes including operative time, blood loss, intraoperative complication, days to solid diet, length of hospital stay, and postoperative complications. Postoperative complications were defined as any condition requiring conservative or surgical treatment occurring within 30 days after operation. Postoperative mortality was defined as death within 30 days of operation. Postoperative complications included anastomotic leakage, bowel obstruction, enterocolitis, intra-abdominal abscess, intra-abdominal bleeding, and pneumonia. Postoperative complications were assessed using the Clavien-Dindo classification (CD) categories [14]. From the pathological records, the depth of invasion was examined at the longest cut section line of the tumor, and lymph node metastasis was examined at the largest cut section of the lymph node. All tissues were examined by expert pathologists. And, long-term outcomes including 5-year overall survival (OS) and 5-year disease-specific survival (DSS) were evaluated.

### Statistical analysis

Quantitative data are given as the median and range. Differences between the three groups were assessed by the chi-square test, Fisher’s exact test, or Mann-Whitney U test as appropriate. Long-term outcomes were compared between each group by log-rank test and are summarized as Kaplan-Meier curves and hazards ratios with 95% confidence intervals. These analyses were carried out using SPSS ver. 24 (SPSS Inc., Chicago, IL, USA). A *P*-value < .05 was considered statistically significant.

## Results

Characteristics of the patients in the three groups are given in Table 1. The average age of the patients in the LAC-E, OC-E, and LAC-NE groups were 84, 84, and 65 years, respectively. There were no differences between LAC-E and OC-E groups in patient characteristics. The LAC-NE group had higher percentage of men and postoperative chemotherapy than the LAC-E group. And, ASA-PS, presence of symptom, and overall comorbidity were significantly higher in the LAC-E group than that in the LAC-NE group.

**Table 1:** Patient characteristics

Factors	LAC-E group	OC-E group	LAC-NE group	LAC-E vs OC-E	LAC-E vs LAC-NE
	(*n* = 85)	(*n* = 25)	(*n* = 358)	*P*-value	*P*-value
Age (years, mean ± SD)	84 ± 4	84 ± 4	65 ± 11	NS	**< .01**
Gender					
M	42	11	227	NS	**< .05**
F	43	14	131		
ASA-PS					
1 or 2	65 (76%)	19 (76%)	341 (95%)	NS	**< .01**
3 or more	20 (24%)	6 (24%)	17 (5%)		
Presence of symptom	61 (72%)	22 (88%)	214 (60%)	NS	**< .05**
Previous abdominal surgery	23 (27%)	6 (24%)	123 (34%)	NS	NS
Comorbidities					
Overall comorbidity	53 (62%)	15 (60%)	163 (46%)	NS	**< .05**
Cardiac disease	15 (18%)	5 (20%)	27 (8%)	NS	**< .01**
Hypertension	33 (39%)	9 (36%)	81 (23%)	NS	**< .01**
Diabetes Mellitus	10 (12%)	3 (12%)	52 (15%)	NS	NS
Respiratory disease	7 (8%)	3 (12%)	14 (4%)	NS	NS
Renal disease	6 (7%)	1 (1%)	10 (3%)	NS	NS
Cerebrovascular disease	5 (6%)	1 (1%)	5 (1%)	NS	**< .05**
Preoperative chemotherapy	2 (2%)	1 (1%)	18 (5%)	NS	NS
Postoperative chemotherapy	8 (9%)	4 (16%)	127 (35%)	NS	**< .01**

*ASA-PS* American Society of Anesthesiologists physical status, *LAC-E* laparoscopic surgery in elderly patients, *LAC-NE* laparoscopic surgery in non-elderly patients, *NS* not significant, *OC-E* open surgery in elderly patients

**Fig. 1:**
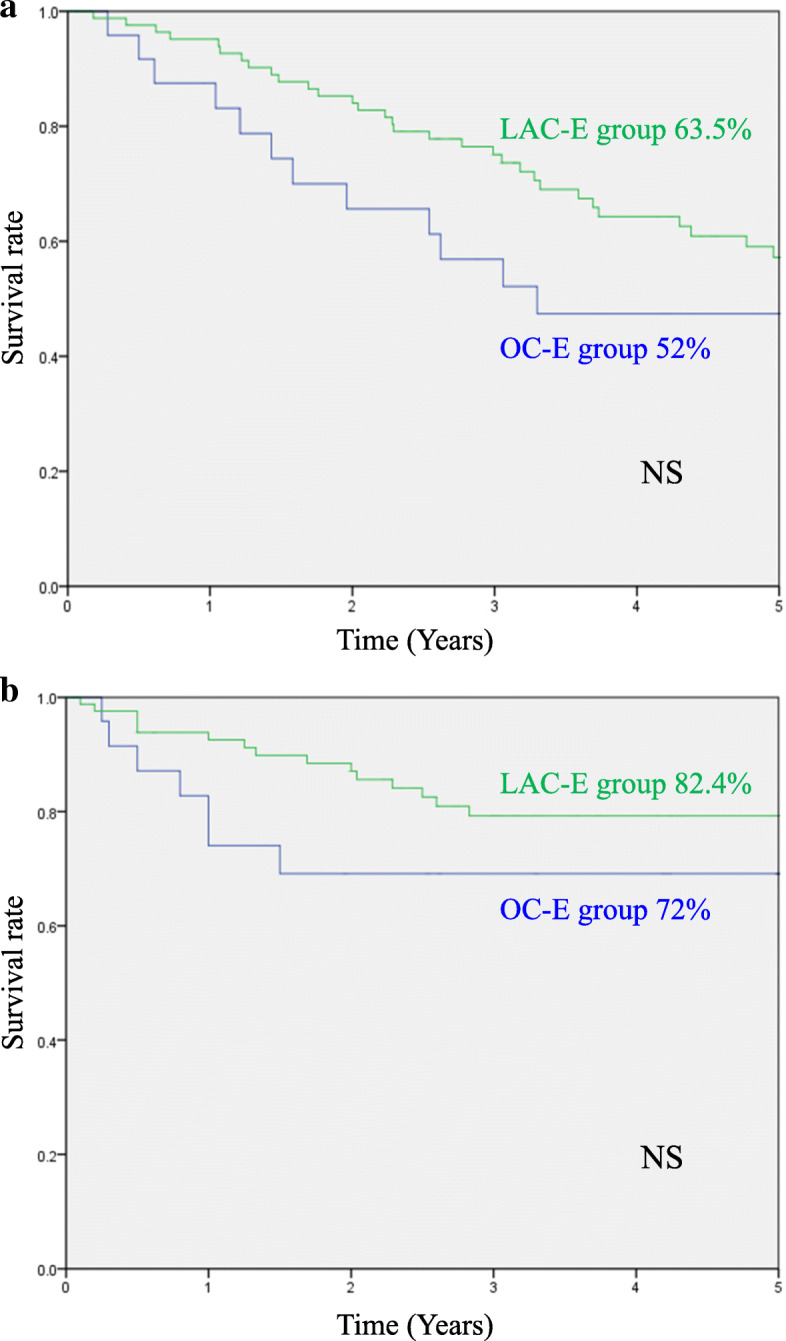
**a** Comparison of 5-year overall survival between LAC-E and OC-E groups. **b** Comparison of 5-year disease-specific survival between LAC-E and OC-E groups

Comparisons between the LAC-E and OC-E groups are summarized in Table 2. Pathological findings including pT stage and pN stage were not statistically different between the two groups. Regarding the short-term outcomes, the amount of blood loss (LAC-E vs. OC-E: 97 vs. 440 g, *p* < .01) and the rate of morbidity (≥CD grade II) (14% vs. 32%, *p* < .05) in the LAC-E group were significantly lower than those in the OC-E group. In the analysis of long-term outcomes, there were no significant differences in 5-year OS (LAC-E vs. OC-E: 63.5% vs. 52%) (Fig. 1a) and 5-year DSS (82.4% vs. 72%) (Fig. 1b) between the two groups.

**Table 2:** Pathological findings and short-term outcomes in the LAC-E and OC-E groups

Factors	LAC-E group	OC-E group	*P*-value
	(*n* = 85)	(*n* = 25)	
**Pathological findings**			
Tumor location			
Right colon	38 (44%)	9 (36%)	NS
Left colon	15 (18%)	6 (24%)	
Rectum	32 (38%)	10 (40%)	
Tumor differentiation			
Well/moderately	75 (88%)	21 (84%)	NS
Poorly/mucinous	10 (12%)	4 (16%)	
Tumor size (mm, mean ± SD)	47 ± 21	67 ± 29	NS
pT stage			
T0-T1	13 (15%)	1 (4%)	NS
T2-T4	72 (85%)	24 (96%)	
pN stage			
N0	50 (59%)	16 (64%)	NS
N1-N2	35 (41%)	9 (36%)	
TNM Stage			
0-II	47 (55%)	16 (64%)	NS
III-IV	38 (45%)	9 (36%)	
**Short-term outcomes**			
Operative time (min, mean ± SD)	249 ± 87	223 ± 111	NS
Blood loss (g, mean ± SD)	97 ± 124	440 ± 740	**< .01**
Intraoperative complication	0	0	NS
Days to solid diet (days, mean ± SD)	4.1 ± 2	4.6 ± 2	NS
Length of hospital stay (days, mean ± SD)	23 ± 39	35 ± 37	NS
Postoperative complication			
Mortality	0	0	NS
Overall morbidity (CD grade 2 or more)	12 (14%)	8 (32%)	**< .05**
Anastomotic leakage	0	0	NS
Bowel obstruction	5 (6%)	3 (12%)	NS
Enterocolitis	3 (4%)	1 (4%)	NS
Intraabdominal abscess	2 (2%)	1 (4%)	NS
Bleeding	0	0	NS
Pneumonia	0	1 (4%)	NS
Others	2 (2%)	2 (8%)	NS

*CD* Clavien-Dindo, *LAC-E* laparoscopic surgery in elderly patients*, NS* not significant, *OC-E* open surgery in elderly patients

**Fig. 2:**
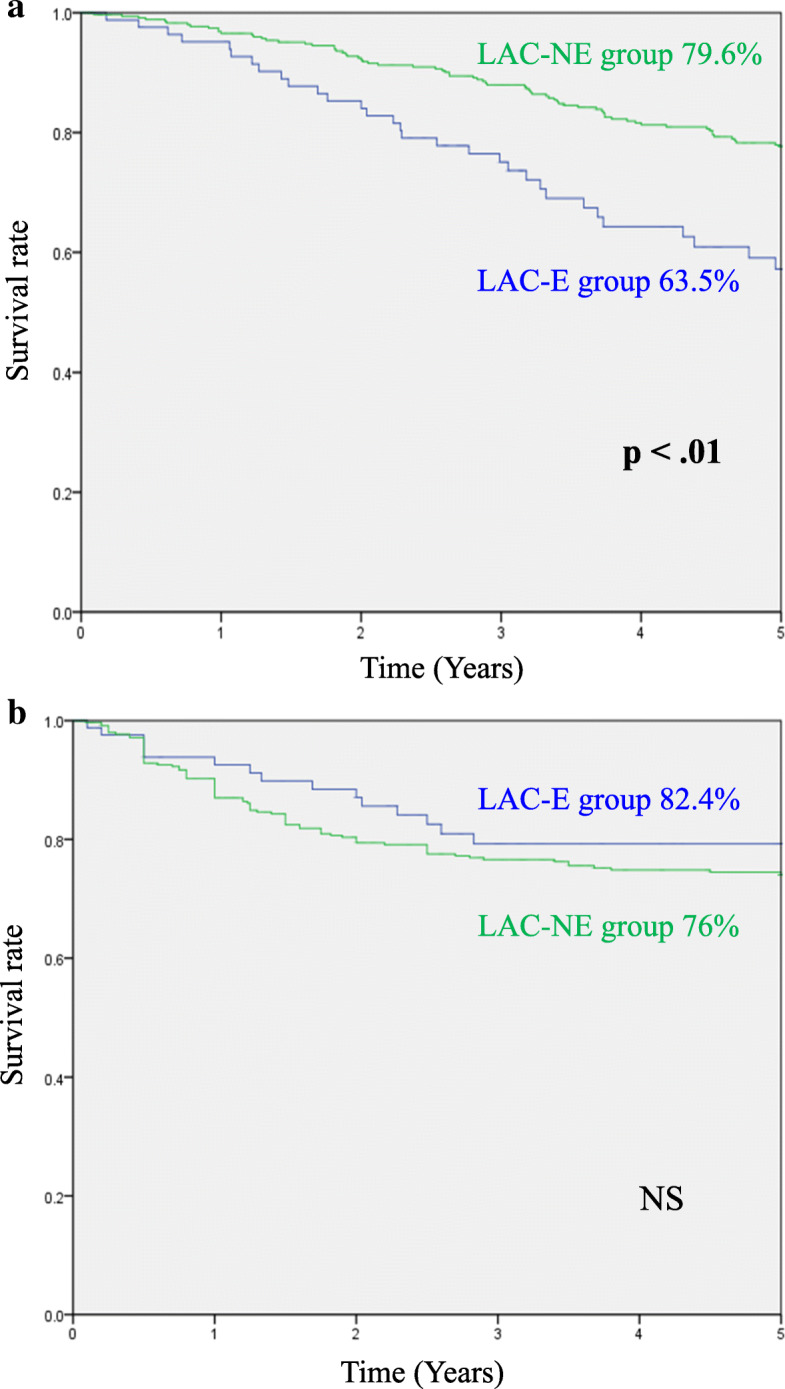
**a** Comparison of 5-year overall survival between LAC-E and LAC-NE groups. **b** Comparison of 5-year disease-specific survival between LAC-E and LAC-NE groups

Comparisons between the LAC-E and LAC-NE groups are summarized in Table 3. The proportions of patients who had pathological tumor stage T0–1 were significantly higher in the LAC-NE group than LAC-E group (LAC-NE vs. LAC-E: 26.2% vs. 15.2%, *p* < .05). Among the short-term outcomes, operation time was shorter in the LAC-E group than LAC-NE group (249 vs. 288 min, *p* < .01). There were no differences in blood loss, the incidence of postoperative complications, days to solid diet, and length of hospital stay between the two groups. In the long-term outcomes, the 5-year OS rate in the LAC-E group was significantly lower than that in the LAC-NE group (63.5% vs. 79.6%, *p* < .01) (Fig. 2a), whereas there was no significant difference between the two groups in 5-year DSS (82.4% vs. 76%) (Fig. 2b).

**Table 3:** Pathological findings and short-term outcomes in the LAC-E and LAC-NE groups

Factors	LAC-E group	LAC-NE group	*P*-value
	(*n* = 85)	(*n* = 358)	
**Pathological findings**			
Tumor location			
Right colon	38 (44%)	116 (32%)	NS
Left colon	15 (18%)	92 (26%)	
Rectum	32 (38%)	150 (42%)	
Tumor differentiation			
Well/moderately	75 (88%)	325 (91%)	NS
Poorly/mucinous	10 (12%)	33 (9%)	
Tumor size (mm, mean ± SD)	47 ± 21	41 ± 20	NS
pT stage			
T0-T1	13 (15%)	94 (26%)	**< .05**
T2-T4	72 (85%)	264 (74%)	
pN stage			
N0	50 (59%)	233 (65%)	NS
N1-N2	35 (41%)	125 (35%)	
TNM Stage			
0-II	47 (55%)	226 (63%)	NS
III-IV	38 (45%)	132 (37%)	
**Short-term outcomes**			
Operative time (min, mean ± SD)	249 ± 87	288 ± 118	**< .01**
Blood loss (g, mean ± SD)	97 ± 124	125 ± 223	NS
Intraoperative complication	0	1	NS
Conversion to open surgery	0	1	NS
Days to solid diet (days, mean ± SD)	4.1 ± 2	4.5 ± 4	NS
Length of hospital stay (days, mean ± SD)	23 ± 39	18.2 ± 16	NS
Postoperative complication			
Mortality	0	0	NS
Overall morbidity (CD grade 2 or more)	12 (14%)	32 (9%)	NS
Anastomotic leakage	0	4 (1%)	NS
Bowel obstruction	5 (6%)	9 (3%)	NS
Enterocolitis	3 (4%)	7 (2%)	NS
Intraabdominal abscess	2 (2%)	6 (2%)	NS
Bleeding	0	2 (0.6%)	NS
Pneumonia	0	0	NS
Others	2 (2%)	4 (1%)	NS

*CD* Clavien-Dindo, *LAC-E* laparoscopic surgery in elderly patients*, NS* not significant, *LAC-NE* laparoscopic surgery in non-elderly patients

## Discussion

In the present study, to clarify the technical and oncological safety of laparoscopic surgery for colorectal cancer in the elderly, we compared the short- and long-term outcomes between the elderly patients in the laparoscopic surgery and open surgery groups, and the non-elderly patients in the laparoscopic surgery group. The results of the comparison between the laparoscopic surgery and open surgery groups of elderly patients showed that the intraoperative blood loss and incidence of postoperative complications were significantly lower in the laparoscopic group, and there were no differences in long-term outcomes between the two groups. When comparing the elderly and non-elderly laparoscopic surgery groups, the operation time was significantly shorter in the elderly group. In terms of long-term outcomes, although the elderly group had shorter OS, there was no difference in DSS between the two groups. These results suggest that laparoscopic colorectal surgery is less invasive than open surgery and is oncologically safe for elderly patients and for younger patients.

As shown in previous reports, laparoscopic colorectal surgery for elderly patients had advantages in short-term outcomes such as intraoperative blood loss, time to normal bowel function, and the length of hospital stay, similar to those in younger patients [15, 16]. However, our results showed that there was no advantage related to length of hospital stay in the laparoscopic group. This finding might have been caused by the lack of a clinical pathway system in our department. Because there was no clinical pathway system into our department, we might not have recommended that elderly patients be discharged from hospital, despite the early recovery of the patients in the laparoscopic group. The fact that there was also no difference in the postoperative course between the elderly and non-elderly patients in our study supports our view.

In this study, the average operation time was about 30 min shorter in the elderly laparoscopic group than non-elderly group. The reason was considered the possibility in which the operation team tried to shorten the operation time as much as possible because of higher ASA-PS and many comorbidities in the elderly laparoscopic group. So far, the operation time has been identified as a risk factor associated with the occurrence of postoperative complications of laparoscopic colectomy. Bailey et al. revealed that the operation time > 3 h was an independent risk factor for infectious complications in patients undergoing a laparoscopic right colectomy [17]. Scheer et al. also reported that colectomies lasting more than 270 min were associated with increased postoperative complications, extension of days to surgical diet, and longer hospital stay [18]. In our study including rectal cancer, it is not clear how shortening the operation time contributed to the incidence of complications and early postoperative recovery in the elderly laparoscopic group. We consider that it will be necessary to evaluate the effect of operative time on the incidence of complication, in order to improve the safety of laparoscopic surgery for elderly patients with colorectal cancer from now on.

In relation to postoperative complications, some of the previous studies in elderly patients have reported the rates of morbidity in laparoscopic and open surgery to be similar [19, 20]. In contrast, other studies have shown that the rate of overall morbidity was lower in laparoscopic surgery than in open surgery [21–25]. Kennedy et al. reported that open surgery was one of the factors associated with an increased risk of complications in multivariate analysis using the database of the American College of Surgeons for elderly patients with colon cancer [26]. We also observed a lower rate of overall morbidity in the laparoscopic group than that in the open group. However, it is still controversial whether laparoscopic colorectal surgery for elderly patients is as safe as it is in non-elderly patients. It is general knowledge that the incidence of postoperative complications causing a serious condition after major digestive surgery is considered to be higher in elderly patients because of the reduced functional potential of their organs and having significant age-related comorbidities such as cardiovascular and pulmonary disease [27–29]. Hermans et al. also reported that the incidence of complications in elderly patients was significantly higher than that in younger patients [30]. However, Tokuhara et al. reported in a prospective cohort study that there were no differences between the elderly and younger patients in the incidence of postoperative complications after laparoscopic colorectal surgery [31]. Kahn et al. found that older age is not independently associated with complications after surgery for colorectal cancer [32]. Our results also showed that the rate of postoperative complications in the elderly patients did not increase compared with that in the non-elderly patients, in spite of the higher status of ASA-PS and the greater number of comorbidities in the elderly patients. These results suggest that laparoscopic colorectal surgery is as equally safe for elderly patients as it is for younger patients.

Regarding the long-term outcomes in this study, no significant differences in OS and DSS were shown between the laparoscopic and open group of elderly patients. These results were consistent with previous randomized control trials in non-elderly patients between laparoscopic and open surgery [33–35]. Niitsu et al. also reported that laparoscopic surgery for elderly colorectal cancer patients with poor PS was not inferior to open surgery in terms of survival [21]. We also believe that laparoscopic surgery for colorectal cancer has equivalent oncological safety to that of open surgery among elderly patients. However, there have been only a few reports comparing long-term outcomes of laparoscopic colorectal surgery between elderly and non-elderly patients. Tokuhara et al. reported that there were no differences in recurrence-free survival and OS between elderly (≥75 years) and non-elderly (< 75 years) patients in a prospective cohort study [31]. Jeong et al. reported that there was no difference between two groups in 3-year disease-free survival, although 3-year OS in the group aged ≥75 years was lower than that of the younger group [36]. The present study also found that only OS in the elderly patients was lower than that in the non-elderly patients. The main reason is that elderly patients die because of diseases other than colorectal cancer. We did not find any significant differences in DSS between the elderly and non-elderly patients, so we believe that laparoscopic colorectal surgery for elderly patients is not inferior to that for non-elderly patients in terms of oncological safety.

There are some limitations in this study. First, this was a single-center, retrospective study. Second, there was selection bias in regard to the choice of the operation method in our study because it differed depending on the time period. Laparoscopic colorectal surgery for elderly patients was indicated in 50% of patients in 2007, whereas this ratio increased to 78% in 2014 in the present study. Lastly, differences in patient characteristics between the elderly and non-elderly patients, such as in TNM stage and perioperative chemotherapy, may also be a problem. It was difficult to apply the case-matching study due to insufficient sample size in this study. In the future, a large-scale multicenter prospective randomized controlled study is necessary.

## Conclusions

In conclusion, laparoscopic colorectal surgery for elderly patients aged ≥80 years was less invasive and technically safer than open surgery and provided a surgical cure as it did for non-elderly patients. From the results of our study, we consider laparoscopic colorectal surgery to be an optimal procedure for elderly patients with colorectal cancer. To confirm our opinion, a multi-center prospective study with larger sample size would be required in the near future. The number of elderly patients with colorectal cancer will continue to increase. It is also necessary to establish the treatment guideline including postoperative care and palliative surgery for elderly patients with colorectal cancer.

## Data Availability

All data generated or analysed during this study are included in this published article.

## Abbreviations

ASA-PS: American Society of Anesthesiologists physical status
CD: Clavien-Dindo classification
OS: Overall survival
DSS: Disease-specific survival
